# The MRI Estimations of Cervical Length and Cervical Volume Correlate With Massive Hemorrhage in Patients With Placenta Accreta Spectrum Disorders: A Case‐Control Study

**DOI:** 10.1155/jp/6250135

**Published:** 2026-06-11

**Authors:** Yongfei Yue, Jun Yan, Ye Song, Xinfang Zhou, Yan Kang, Min Yuan

**Affiliations:** ^1^ Department of Obstetrics, The Affiliated Suzhou Hospital of Nanjing Medical University, Suzhou Municipal Hospital, Suzhou, Jiangsu, China, njmu.edu.cn

**Keywords:** cervical volume, massive hemorrhage, MRI, placenta accreta spectrum

## Abstract

**Objective:**

The aim of this study is to evaluate the predictive value of two magnetic resonance imaging (MRI) parameters for massive hemorrhage (MH) in pregnancies with placenta accreta spectrum (PAS) disorders.

**Methods and Materials:**

This case‐control study included 174 patients who underwent MRI for placental assessment. MRI images were independently reviewed by two experienced radiologists blinded to all clinical data. Three‐dimensional (3D) models of the cervix were reconstructed, and cervical volume was measured using 3D Slicer software. Multivariate logistic regression analysis was performed to identify risk factors for MH. The predictive performance of cervical length and cervical volume was compared using the area under the receiver operating characteristic curve (AUC).

**Results:**

Compared to patients without MH, those with MH had neonates with significantly lower birth weight (*p* < 0.001) and were delivered at an earlier mean gestational age (*p* < 0.001). The MH group also experienced longer operative times (*p* < 0.001), greater estimated blood loss (*p* < 0.001), and significantly higher rates of ICU admission and hysterectomy. In predicting MH, cervical volume demonstrated superior performance to cervical length (AUC: 0.85 vs. 0.81). The predictive accuracy was further enhanced when both parameters were combined (AUC = 0.906).

**Conclusions:**

Shorter cervical length and smaller cervical volume are associated with an increased risk of MH during cesarean section in pregnancies complicated by PAS. These MRI parameters may serve as useful adjuncts for preoperative risk stratification and hemorrhage prediction.

## 1. Introduction

The placenta accreta spectrum (PAS) is primarily attributed to failed normal decidualization, leading to the penetration of placental chorionic villi into the myometrium, serosa, or adjacent organs. This serious obstetric complication is classified into three types based on the depth of invasion: placenta accreta, increta, and percreta [1, 2]. The most common risk factors include prior cesarean delivery and placenta previa [3]. The rising incidence of PAS—now affecting approximately 0.01%–1.1% of pregnancies—parallels the increasing rate of cesarean sections worldwide [4]. Unpredictable, life‐threatening hemorrhage remains a major cause of maternal morbidity and mortality [5]. Despite this, validated models for predicting intraoperative hemorrhage in PAS remain scarce.

Several observational studies have investigated the association between cervical length and the risk of hemorrhage in pregnancies complicated by PAS [6, 7]. A shortened cervix may limit contraction of the lower uterine segment and complicate surgical hemostasis [8]. While cervical length measurement is simple and readily obtainable, three‐dimensional (3D) volumetric assessment offers more detailed anatomical information and may enhance the prediction of intraoperative hemorrhage compared to conventional two‐dimensional (2D) evaluation.

Ultrasound remains the primary imaging modality for diagnosing PAS. However, its diagnostic accuracy may be compromised by a posterior placenta or increased maternal abdominal wall thickness. Although transvaginal ultrasound can help overcome these limitations, it is not routinely performed in all settings. MRI offers superior spatial resolution and allows comprehensive assessment of placental invasion depth, with numerous studies demonstrating high sensitivity and specificity for PAS diagnosis [9]. We hypothesized that cervical length and cervical volume, as measured by MRI, are independent predictors of massive hemorrhage (MH) in PAS and that their combination provides superior risk stratification. Specifically, we aimed to evaluate the predictive utility of these two parameters for postpartum hemorrhage in this population.

## 2. Materials and Methods

### 2.1. Study Population

This study was a case‐control design by screening the patients with PAS disorders from January 2016 to October 2023 and was approved by the Ethics Committee of the Affiliated Suzhou Hospital of Nanjing Medical University (K‐2022‐015‐K01). Considering the anonymous patient data, informed consents were waived by the committee. Our study adhered to the Declaration of Helsinki.

**Figure 1:**
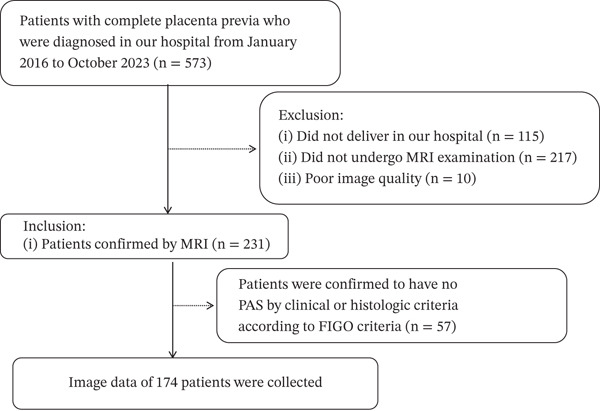
Flow chart of patients with complete placenta previa included in the study.

The inclusion criteria were (1) singleton pregnancy, (2) at least one previous cesarean section, and (3) suspected PAS on ultrasound and MRI at 28–32 weeks of gestational age [10]. Patients with incomplete images and antenatal examination information were excluded. The use of prophylactic balloon occlusion and emergency surgeries was ruled out. All patients had regular prenatal ultrasonic testing and underwent an MRI before the C‐section. Finally, 174 patients were incorporated into the study, and all cases were confirmed as PAS by placental pathological examinations (Figure 1).

### 2.2. Definitions and References of Maternal Complications

PAS disorders were stratified into levels by placenta increta, placenta percreta, and placenta accreta, including clinical criteria and histologic criteria [11]. This study defined MH as > 2000 mL during 24 h after cesarean deliveries [12]. Postpartum blood loss was estimated through the volume method and the weighing method. In this study, we carefully reviewed all the cases and performed transabdominal sonography scores based on the study by Jauniaux et al. [13], with 1 point for each item, for a maximum of 7 points. The seven typical ultrasound signs of PAS include (1) loss of the “clear zone,” (2) myometrial thinning, (3) bladder‐wall interruption, (4) placental bulge, (5) uterovesical hypervascularity, (6) placental lacunae, and (7) bridging vessels.

### 2.3. MR Assessment of Cervical Length and Volume

**Figure 2:**
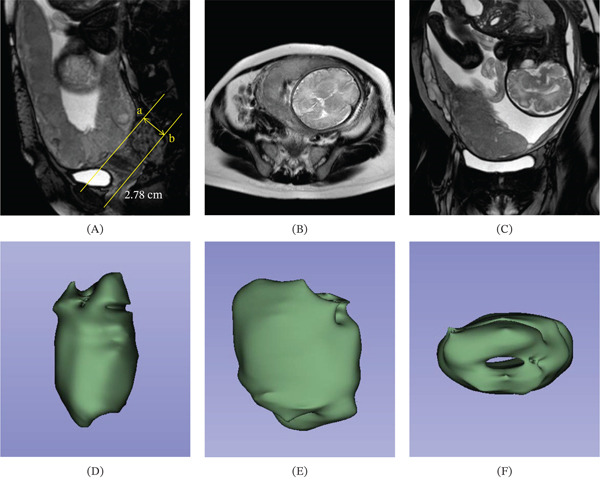
The cervical length was measured, and the cervical volume was reconstructed. (A) Cervical length was measured between lines a and b in a sagittal MRI image (2.78 cm). (B) Axial placenta image of a patient with PAS by MRI. (C) Coronal placenta image of a patient with PAS by MRI. (D) Sagittal image of the three‐dimensional cervix generated by 3D Slicer. (E) Coronal image of the three‐dimensional cervix generated by 3D Slicer. (F) Axial image of the three‐dimensional cervix generated by 3D Slicer.

MR imaging was performed in a 3.0 T MRI system (Siemens Medical Solutions, Erlangen, Germany) with an identical protocol using a pelvic phased‐array coil, and any intravenous injections were not used. T1‐ and T2‐weighted images in the axial, sagittal, and coronal orientations were obtained. Imaging parameters were a repetition time of 700 msec, an effective echo time of 87 msec, a bandwidth of 698 Hz/px, a 432∗432 matrix over a field of view of 380∗380 mm, and a 5 mm slice thickness. Cervical length was measured between lines a and b in sagittal images (Figure 2). Cervical volume, as measured in millimeters, was calculated with 3D Slicer software (Version 5.2.1, available at https://www.slicer.org). Cervical volume reconstructions from three different perspectives are shown in Figure 2. All MRI data were analyzed in consensus by two experienced radiologists. After the identification of the cervical parameters, another radiologist completed the volume measurement. Radiologists were blinded to the clinical diagnosis and treatment.

### 2.4. Data Collection and Analysis

The baseline characteristics, including maternal age, BMI, and reproductive history, were collected. Researchers also recorded the following delivery information: gestational age at delivery, neonatal birth weight, operation time, intraoperative blood loss, blood transfusion, intensive care unit (ICU) admission, and hysterectomy.

All statistical analyses were analyzed by SPSS 23.0 statistics software (SPSS Inc., Chicago, IL, United States). The graphs were performed using GraphPad Prism 8.0 software. The variables were expressed as mean ± standard deviation or median (interquartile range) according to the distribution of the data. The difference of variables was compared with the *T*‐test, Mann–Whitney *U* test, chi‐square test, or Fisher′s exact test. All reported *p* values were with two tails. The agreement between observers was assessed using the kappa test. A kappa value of 0.75 signified strong consistency, while a kappa value between 0.4 and 0.75 indicated moderate consistency. The receiver operating characteristic (ROC) curve was used to evaluate the diagnostic value of different cervical parameters. Sensitivity and specificity analyses were conducted by excluding outlier data. *p* < 0.05 was considered to be statistically significant.

## 3. Results

A total of 174 patients were included in this study. Baseline characteristics of the study population are presented in Table 1. Intraoperatively, 81 patients were diagnosed with PAS disorders, including 33 cases of accreta, 28 of increta, and 20 of percreta. All patients underwent cesarean section delivery. No statistically significant differences were observed between groups with respect to maternal age, BMI, gravidity, parity, history of previous cesarean delivery, or gestational age at the time of MRI examination.

**Table 1:** Clinical features of the study groups (*n*/%).

Characteristic	Patients with MH (*n* = 81)	Patients without MH (*n* = 93)	Statistic (*t*//*Z*/*χ* ^2^)	*p* value
Maternal age	32.04 ± 4.06	31.20 ± 4.17	1.329	0.185
BMI (kg/m^2^)	25.85 ± 3.19	25.69 ± 3.89	0.301	0.764
Gravidity	3 (2.4)	3 (2.4)	1.200	0.230
Parity	2 (1.2)	2 (1.2)	0.625	0.532
Number of abortions	1 (1.2)	1 (1.2)	1.331	0.183
Number of previous cesarean deliveries	1 (1.2)	1 (1.2)	0.552	0.581
Previous history of placenta previa	9 (11.11)	5 (5.38)	1.924	0.165
Gestational age at MRI (weeks)	33.33 ± 2.12	33.60 ± 1.76	0.923	0.358
Prenatal vaginal bleeding	27 (33.33)	25 (26.88)	0.860	0.354
Transabdominal sonography score	5.35 ± 1.84	3.42 ± 1.24	8.202	< 0.001
Gestational age at delivery (weeks)	35.23 ± 1.48	35.95 ± 1.14	3.621	< 0.001
Neonatal birthweight (g)	2415.56 ± 365.99	2568.53 ± 352.05	2.807	0.006
Operation time (min)	92.30 ± 26.59	79.51 ± 28.29	3.058	0.003
Intraoperative blood loss (mL)	2377.70 ± 657.17	1214.01 ± 364.06	14.688	< 0.001
Blood transfusion (mL)	1802.10 ± 634.35	751.51 ± 396.05	13.277	< 0.001
ICU admission	17 (20.99)	7 (7.53)	6.597	0.010
Hysterectomy	6 (7.41)	1 (1.08)	4.496	0.034
The type of PAS			14.452	0.001
Accreta	33 (40.74)	56 (60.22)		
Increta	28 (34.57)	32 (34.41)		
Percreta	20 (24.69)	5 (5.38)		

Patients with MH had a significantly lower mean gestational age at delivery compared to those without MH (35.23 ± 1.48 vs. 35.95 ± 1.14 weeks; *p* < 0.001). The MH group also experienced longer operative times (92.30 ± 26.59 vs. 79.51 ± 28.29 min; *p* = 0.003) and greater intraoperative blood loss (2377.70 ± 657.17 vs. 1214.01 ± 364.06 mL; *p* < 0.001). Regarding adverse clinical outcomes, 17 patients (20.99%) required ICU admission, six (7.41%) underwent emergency hysterectomy, and the MH group received significantly more blood transfusions (1802.10 ± 634.35 vs. 751.51 ± 396.05 mL; *p* < 0.001) due to massive bleeding and hemodynamic instability.

Interobserver agreement between the two radiologists for the measurement of cervical length and volume was almost perfect, with kappa values exceeding 0.75 (Table 2). Table 3 presents the odds ratios for MH in relation to cervical length and volume. Patients with a small cervical volume (< 20.00 cm^3^) had a significantly higher odds ratio for MH (OR = 6.384; 95% CI: 2.517–9.657; *p* < 0.001).

**Table 2:** Interobserver reliability of magnetic resonance imaging (MRI) in the measurement of MRI features.

MRI features	Either	Both	Agree	Kappa	Interpretation
Cervical length	93 (53.448)	85 (48.851)	95.402	0.908	Almost perfect
Cervical volume	95 (54.598)	88 (50.575)	95.977	0.919	Almost perfect

*Note:* Presented here as no. (percentage). Number (and proportion) of studies in which either radiologist or both radiologists reported a finding. Percentage of studies in which they agreed.

**Table 3:** Multivariate logistic regression analysis of risk factors for patients with MH.

Variable	Multivariate analysis	
	OR (95% CI)	*p*
Maternal age (> 35)	1.265 (0.768–1.739)	0.013
Gravidity (> 3)	1.187 (0.863–1.655)	0.036
Parity (> 2)	1.392 (0.892–1.892)	0.029
History of abortions	1.857 (1.394–2.303)	0.022
History of previous cesarean delivery	2.305 (1.851–2.920)	< 0.001
History of placenta previa	3.537 (2.593–5.704)	< 0.001
Prenatal vaginal bleeding	0.950 (0.514–1.380)	0.082
Gestational age at delivery (> 35 weeks)	1.493 (0.705–1.903)	0.017
Neonatal birth weight (> 2500 g)	1.564 (0.858–2.073)	0.008
Cervical length (< 3 cm)	3.512 (1.836–6.927)	< 0.001
Cervical volume (< 20 cm^3^)	6.384 (2.517–9.657)	< 0.001

*Note:* After adjusting for maternal age, gravidity, parity, history of abortions, history of previous cesarean delivery, history of placenta previa, gestational age at delivery, and neonatal birth weight, cervical length (< 3 cm) and cervical volume (< 20 cm^3^) showed a significant correlation with MH (*p* < 0.001).

**Figure 3:**
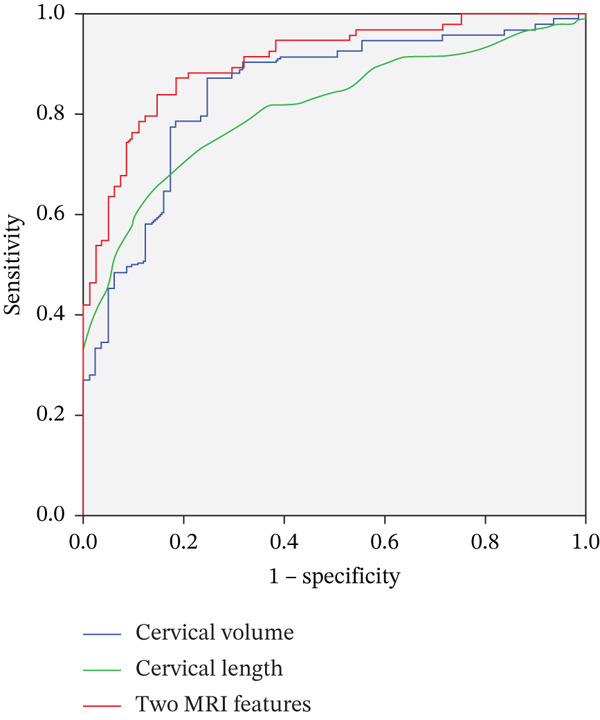
Receiver operating characteristic (ROC) curve of different magnetic resonance imaging (MRI) features in patients with placenta accreta spectrum (PAS) disorders.

The optimal cutoff values for cervical length and cervical volume were identified as 30 mm and 20 cm^3^, respectively, based on ROC analysis (Table 4). Using a cervical length of < 30 mm to predict high risk of MH yielded a sensitivity of 86.53%, specificity of 83.94%, PPV of 82.43%, and NPV of 87.74%, with an area under the curve (AUC) of 0.81. For cervical volume < 20 cm^3^, the sensitivity, specificity, PPV, and NPV were 91.53%, 83.61%, 82.94%, and 84.63%, respectively, with an AUC of 0.85 (Figure 3). When cervical length and volume were combined, the AUC increased to 0.906.

**Table 4:** Receiver operating characteristic analyses for prediction of massive hemorrhage based on cervical length and cervical area.

Variable	Cut‐off	Sensitivity (%) (95% CI)	Specificity (%) (95% CI)	PPV (%)	NPV (%)	*p*
Cervical length (mm)	28	81.11 (73.55–87.62)	88.33 (80.58–93.39)	85.82	84.30	< 0.001
Cervical length (mm)	30	86.53 (78.81–90.95)	83.94 (77.23–90.65)	82.43	87.74	< 0.001
Cervical length (mm)	32	83.52 (76.81–88.76)	84.08 (79.27–92.85)	82.04	85.41	< 0.001
Cervical volume (cm^3^)	18	84.46 (79.25–89.90)	87.21 (81.17–91.92)	85.19	86.56	< 0.001
Cervical volume (cm^3^)	20	91.53 (83.91–94.06)	83.61 (77.88–89.05)	82.94	84.63	< 0.001
Cervical volume (cm^3^)	22	88.57 (80.54–91.93)	86.35 (80.90–90.38)	84.96	82.47	< 0.001

*Note:* PPV, positive predictive value, is the proportion of individuals with a positive test result who truly have massive hemorrhage; NPV, negative predictive value, is the proportion of individuals with a positive test result who truly have massive hemorrhage.

**Figure 4:**
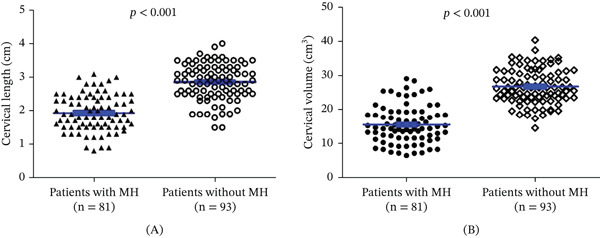
The difference in cervical length and volume between patients with and without MH. (A) Cervical length in patients with and without MH. (B) Cervical volume in patients with and without MH.

**Figure 5:**
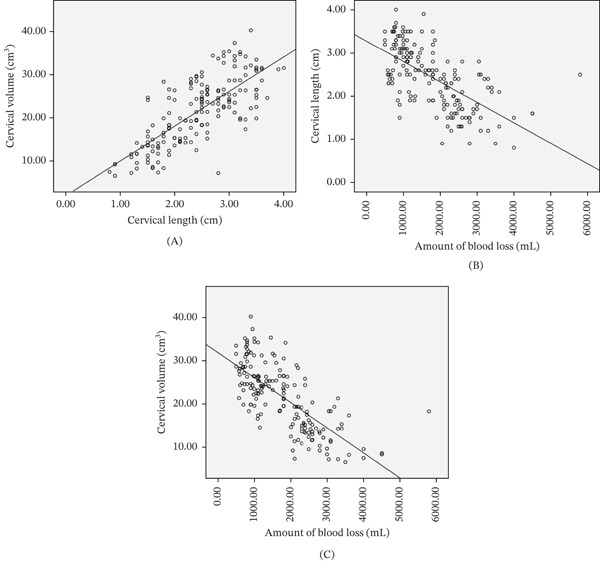
Association among cervical length, cervical volume, and amount of blood loss. (A) Association between cervical length and cervical volume in PAS patients (*r* = 0.748). (B) Association between cervical length and amount of blood loss in PAS patients (*r* = −0.624). (C) Association between cervical area and amount of blood loss in PAS patients (*r* = −0.723). Positive value of *r* indicates positive correlation, and a negative value indicates negative correlation.

As shown in the subcolumn graphs (Figure 4), patients with MH demonstrated a significant difference in both cervical length and volume compared to those without MH. Furthermore, the scatter diagram (Figure 5) illustrated that both cervical length and volume exhibited a negative correlation with the volume of intraoperative blood loss. The findings of this study indicate that cervical measurements are strong predictors of MH in patients with PAS disorders, with an adjusted *R* ^2^ of 0.86.

## 4. Discussion

The prevalence of PAS disorders has risen in parallel with the increasing rates of primary and repeated cesarean deliveries. Elective cesarean delivery remains the standard treatment, with intraoperative bleeding control posing a major surgical challenge [14, 15]. Previous studies have suggested that a shorter cervical length is associated with a higher risk of MH [6, 7, 16, 17]. While ultrasonography is the primary modality for diagnosing PAS and assessing cervical morphology, MRI offers superior soft tissue contrast and multiplanar capabilities, allowing for a more detailed delineation of the uteroplacental interface. Therefore, this retrospective study was aimed at investigating the correlation between MRI‐derived cervical parameters (length and volume) and the risk of MH in patients with PAS.

Adverse perinatal outcomes are strongly correlated with the depth of trophoblastic invasion. Our results corroborate previous findings, demonstrating that the greater the depth of myometrial invasion, the higher the complexity of the surgical procedure and the greater the risk of severe complications, including intraoperative hemorrhage, peripartum hysterectomy, and maternal death [4, 18].

Our findings demonstrate that both cervical length and volume are independently and negatively associated with the risk of MH in PAS. Specifically, a cervical length of less than 3.0 cm was associated with a 3.51‐fold increased risk of MH, while a cervical volume below 20.0 cm^3^ conferred a more than sixfold higher risk. These results are consistent with previous reports, although the reported threshold values vary. For instance, in a study of PAS complicated by placenta previa, Huang et al. found that a cervical length of greater than 33 mm decreased the hemorrhage risk by 44% [8], whereas another study identified a threshold of 25 mm [15]. Our findings on cervical volume provide a novel contribution to this area of investigation.

Several hypotheses have been proposed to explain the association between the cervix and bleeding in PAS, including uterine atony, surgically difficult hemostasis, depletion of clotting factors from antepartum hemorrhage, and the presence of aberrant vasculature [19–22]. However, these mechanisms do not directly translate into a quantitative clinical tool. To address this gap, we evaluated a novel method focusing on 3D cervical morphology. Our findings demonstrate that cervical volume is a superior predictor of MH compared to cervical length alone (AUC: 0.85 vs. 0.81). Moreover, the combination of both parameters yielded the highest predictive accuracy, with an AUC of 0.906, offering enhanced sensitivity and specificity for identifying patients at risk for MH during cesarean section.

Yilmaz et al. demonstrated that cervical volume increases with advancing gestation and typically decreases to approximately 15.3 cm^3^ postpartum [23]. In the context of PAS, we postulate that pathologic pelvic adhesions and abnormal tissue proliferation may impede this physiological increase in cervical volume. A persistently small cervix may therefore serve as a marker of underlying severe pathology and an associated higher risk of excessive bleeding. To further elucidate this relationship, well‐designed longitudinal cohort studies with larger sample sizes are needed to investigate the correlation between cervical volume and objective measures of abnormal vascularization. Such research could refine the predictive value of cervical volume for MH in PAS.

Our findings are consistent with a recent study by Bucak et al. [24], who proposed a cervical length‐based algorithm for delivery timing in PAS and demonstrated that shorter cervical length (< 25 mm) was associated with significantly earlier delivery (34+1 vs. 35+5 weeks) and higher rates of urgent intervention. Together, these converging lines of evidence support the emerging concept that cervical parameters—both length and volume—may serve not only as predictors of intraoperative hemorrhage but also as objective markers to guide individualized delivery timing. Incorporating such measurements into clinical decision‐making could help identify patients who require earlier scheduled delivery versus those who may safely await later gestation, potentially reducing the need for emergent surgery and improving perioperative outcomes.

To our knowledge, this is the first study to systematically evaluate the association between cervical volume and MH in PAS and to concomitantly compare the predictive performance of two MRI‐derived cervical parameters. Our findings establish cervical volume, particularly in combination with cervical length, as a feasible and promising imaging biomarker for preoperative risk stratification. These results have direct clinical implications, facilitating more informed preoperative planning and the formulation of individualized perioperative management strategies for pregnancies complicated by PAS.

### 4.1. Strengths and Limitations

The findings of this study are strengthened by the inclusion of data from a large, tertiary maternal care center, which serves as the primary referral center for the region, enhancing the representativeness of the cohort. Furthermore, potential observer bias was mitigated by having all MRI images independently reviewed by experienced radiologists who were blinded to clinical outcomes and treatment details. However, the retrospective design of this study is a limitation, and our findings warrant validation in prospective cohorts. Future research with larger, multicenter samples is also necessary to confirm the predictive value of cervical volume and to establish its generalizability.

## 5. Conclusion

A shortened cervix and reduced cervical volume on MRI are strong indicators of an elevated risk for MH during cesarean delivery. Routine evaluation of these parameters could serve as an effective warning system, prompting obstetricians to implement enhanced safety precautions during surgery.

## Author Contributions

Min Yuan and Yongfei Yue performed the literature review, study design, and manuscript writing. Yongfei Yue also supervised the research project. Jun Yan, Ye Song, and Yan Kang performed the data collection. Xinfang Zhou provided statistical analysis and medical consultation.

## Funding

This study was funded by the Gusu Health Talent Plan (GSWS2023055) and Suzhou Science and Technology Development Plan (grant number SYW2025052).

## Disclosure

All authors read and approved the final manuscript. Our research has a preprint at Research Square: Yongfei Yue, Jun Yan, Ye Song et al. The MRI Estimations of Cervical Length and Cervical Volume Correlate With Massive Hemorrhage in Patients With Placenta Accreta Spectrum Disorders, August 16, 2024, PREPRINT (Version 1) (10.0.82.211/rs.3.rs-4659075/v1).

## Conflicts of Interest

The authors declare no conflicts of interest.

## Data Availability

The data that support the findings of this study are available from the corresponding author upon reasonable request.
